# Chronic Stress and Glucocorticoids: From Neuronal Plasticity to Neurodegeneration

**DOI:** 10.1155/2016/6391686

**Published:** 2016-03-10

**Authors:** Sheela Vyas, Ana João Rodrigues, Joana Margarida Silva, Francois Tronche, Osborne F. X. Almeida, Nuno Sousa, Ioannis Sotiropoulos

**Affiliations:** ^1^Laboratory of Gene Regulation and Adaptive Behaviors, Department of Neuroscience Paris Seine, INSERM U1130, CNRS UMR 8246, Université Pierre et Marie Curie, Paris Cedex 05, France; ^2^Life and Health Sciences Research Institute (ICVS), School of Health Sciences, University of Minho, Campus de Gualtar, 4710-057 Braga, Portugal; ^3^ICVS/3B's-PT Government Associate Laboratory, Guimarães, Braga, Portugal; ^4^Max Planck Institute of Psychiatry, Kraepelinstrasse 2-10, 80804 Munich, Germany

## Abstract

Stress and stress hormones, glucocorticoids (GCs), exert widespread actions in central nervous system, ranging from the regulation of gene transcription, cellular signaling, modulation of synaptic structure, and transmission and glial function to behavior. Their actions are mediated by glucocorticoid and mineralocorticoid receptors which are nuclear receptors/transcription factors. While GCs primarily act to maintain homeostasis by inducing physiological and behavioral adaptation, prolonged exposure to stress and elevated GC levels may result in neuro- and psychopathology. There is now ample evidence for cause-effect relationships between prolonged stress, elevated GC levels, and cognitive and mood disorders while the evidence for a link between chronic stress/GC and neurodegenerative disorders such as Alzheimer's (AD) and Parkinson's (PD) diseases is growing. This brief review considers some of the cellular mechanisms through which stress and GC may contribute to the pathogenesis of AD and PD.

## 1. Introduction

Stress is broadly defined as an actual or anticipated threat of well-being or disruption of organism homeostasis [1]. Although the sensing and reaction to stress evolved to promote adaptation, modern workstyles and lifestyles represent challenges that render individuals susceptible to physical and mental disorders [2–5]. Multiple factors influence an individual's ability to cope with stress, for example, early life experiences, gender, or personality traits. Both vulnerability and resilience may be determined by genetic and epigenetic (gene environmental interactions) background [5–9].

Since the discovery of the communication between hypothalamus and pituitary in early 70s that opens a new window in our understanding of the brain-body communication, there are plethora of studies describing the high biological significance of stress and its responses which enables various adaptive processes to changing conditions. The most easily measureable and critical physiological response to stress involves the release of glucocorticoids (glucocorticoids, GCs). These hormones are synthesized and secreted into systemic circulation from the adrenal glands following stimulation by the anterior pituitary hormone adrenocorticotropic hormone (ACTH) [1]. The release of ACTH itself is increased in response to the secretion of corticotropin-releasing hormone (CRH) and arginine vasopressin (AVP) from neurons in the hypothalamic paraventricular nucleus (PVN). Together, the hypothalamus, pituitary, and adrenal glands constitute the so-called hypothalamo-pituitary-adrenal (HPA) axis, which plays an essential role in the adaptive response to psychogenic (e.g., fear) and physical (e.g., cellular lesion or pathogen invasion) stressors. The adaptive responses that are initiated by GCs occur in multiple tissues and involve alterations in numerous physiological (e.g., metabolic, cardiovascular, and immune) as well as behavioral (e.g., emotion, cognition, and motor) processes [1, 10–12]. Normally, GC-driven negative feedback mechanisms at the different levels of the HPA axis serve to normalize GC secretion and restore homeostasis; however, and depending on the type, duration, and intensity of the stressful stimulus, GC hypersecretion may persist and become a potential threat for health [1].

There is now abundant evidence that GCs can exert profound modulatory effects on a variety of brain functions from early development through to late life [12]. Their actions are mediated by two receptors: the mineralocorticoid receptor (MR) and glucocorticoid receptor (GR), which belong to the superfamily of nuclear receptors that act as transcription modulators [13, 14]. In the brain, GR is ubiquitously expressed, whereas MR expression is more restricted to just a few structures (hippocampus, locus coeruleus, amygdala, prefrontal cortex, and nucleus of the solitary tract, as well as PVN neurons). MR is also present in nonneuronal cells, namely, in glia and epithelial cells of the choroid plexus and ependyma [15].

Binding assays using ^3^[H] corticosterone have shown the MR has a 10-fold higher affinity (*K*_*d*_ = 0.5 nM) for GC compared to GR (*K*_*d*_ = 5 nM), which means that, at basal GC levels, MR is occupied and activated [16] whereas GR is only activated when GC levels reach a certain level, for example, during the circadian peak of GC secretion and during stress [17]. Importantly, brain MR and GR both respond to the same endogenous ligand (cortisol in humans and larger mammals, corticosterone in rodents); further, MR and GR were reported to colocalize in the same pyramidal and granular neurons of the hippocampus [17]. Given the GR and MR colocalization and relatively small difference in their affinity for endogenous GCs, the question arises as to whether they regulate distinct genes and/or coregulate transcription by heterodimerization. Heterodimerization of GR and MR was shown with high concentration of GC (stress level) in the nuclei of cultured hippocampal neurons. Moreover, evidence suggests that their cellular responses through regulation of distinct gene expression (as homodimers) depend strongly upon specific recruitment of coregulators [18, 19].

**Figure 1:**
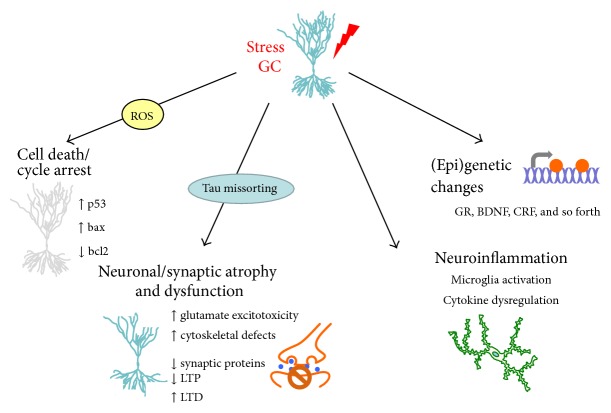
Cellular targets and actions of chronic stress mediated by glucocorticoid receptors. This schema depicts some cellular targets and mechanisms that are targeted by glucocorticoids (GCs), whose actions are mediated by glucocorticoid receptors (GR). GCs are secreted under conditions of stress; neuronal damage and brain pathologies are a common consequence of persistently elevated GC secretion. GC can trigger mitochondrial dysfunction and the apoptotic machinery, as well as cell cycle arrest and cell death. In addition, stress/GC may induce neuronal atrophy and synaptic dysfunction/loss by stimulating hyperphosphorylation of the cytoskeletal protein Tau, thus disturbing the integrity of the cytoskeleton and missorting Tau at synapses. Together, these latter events may eventually result in the degradation of synaptic proteins and receptors and consequently, synaptic plasticity. Stress and GC are also established as modulators of microglial activation and neuroinflammatory processes. Lastly, accumulating evidence indicates that stress and GC can influence neuronal structure and function through epigenetic mechanisms.

Synthetic GCs (e.g., dexamethasone, methylprednisolone) are routinely used in clinical situations due to their powerful anti-inflammatory and immunosuppressive actions. However, a growing body of evidence suggests that high GC exposure in early life can adversely program the HPA axis and increase the susceptibility to develop metabolic, neuropsychiatric, and neurodegenerative disorders [5, 20, 21]. In addition, there is now ample experimental evidence where elevated GC levels and prolonged exposure to stressful conditions induce structural remodeling of neurons with synaptic loss as well as alterations in glial functions, which are frequently maladaptive [22]; see also Figure 1. In this brief review we discuss some of current knowledge about cellular targets and mechanisms through which stress and altered GC levels trigger changes in the brain that may lead towards the development and progression of neurodegenerative pathologies such as Alzheimer's (AD) and Parkinson (PD) disease.

## 2. From Stress-Driven Brain Programming to Neurodegenerative Pathologies

In addition to nongenomic mechanisms that are still incompletely identified [23], chronic stress and GC levels most likely influence neuronal function and connectivity by activating GR-mediated transcription. GRs are normally located in the cytoplasm in association with chaperone proteins such as the heat shock proteins Hsp90 and 70 and the immunophilins FKBP51 and FKBP52. Upon GC binding, conformational change of the GR-chaperone complex results in nuclear translocation of the GR [24, 25]. In the nucleus, GR binds to specific regions of DNA, which possess glucocorticoid response elements (GRE) within the promoters of target genes, leading to cell-type and context-dependent gene expression [26–28]. Transcriptional regulation by GR may occur by (a) direct binding of GR homodimers to GRE within DNA sequences to stimulate transcription, for example,* mitogen-activated protein kinase phosphatase-1* gene; (b) direct binding to negative GRE elements to repress transcription; the gene encoding the prohormone from which ACTH is derived (proopiomelanocortin,* POMC*),* CRH, *and the* CRH receptor* genes are examples of negatively regulated genes; and (c) trans-repression or “tethering,” that is, association with other transcriptional factors that inhibit the transcriptional activity of GR. In the brain, identification of GR-modulated genes is difficult due to the anatomical complexity and cellular heterogeneity. Nevertheless, transcriptomic studies in the hippocampus have identified functional classes of GR target genes which include genes coding for neurotransmitter catabolism, neurotrophic factors and their receptors, signal transduction, energy metabolism, and cell adhesion [29].

In addition to altering gene expression, growing evidence suggests that epigenetic mechanisms represent a means through which stress and GCs can leave long-lasting “memories” of past experiences which, in turn, contributes to shaping the organism's physical and mental health trajectory [21, 30, 31]; see Figure 1. Broadly, epigenetics refers to stable changes in the regulation and/or function of DNA, RNA, and/or proteins that do not involve alterations of their primary sequences. Two well-known examples of epigenetic marks induced by environmental stimuli (e.g., stress) are DNA methylation and histone modification. The first evidence of epigenetic programing in the brain by early life adversity showed that poor maternal care in rats leads to methylation of exon 1_7_ in the* GR *promoter, being accompanied by aberrant behaviors and altered HPA axis responses during adulthood [32, 33]. Subsequently, similar mechanisms were reported in humans who had experienced childhood adversity [34] and in infants born to depressed mothers [35]. The earlier studies in rats were replicated in mice in paradigms of prenatal GC exposure and early postnatal stress; we showed that these pre- and postnatal manipulations resulted in epigenetic modifications of the promoters of neurotransmitter (*dopamine receptor 2*) [36],* GR*, and various GR target genes [37, 38] with long-lasting maladaptive behavioral consequences.

Recent studies also suggest that early life events (e.g., intrauterine infections, maternal stress, and poor maternal and perinatal nutrition) may play a role in the onset of Alzheimer's disease (AD), an age-related neurodegenerative disorder characterized progressive memory and cognitive deficits [39]. From this perspective, AD is probably not determined by a single etiologic factor but results from the interplay between genetic and environmental factors throughout life, possibly explaining why monozygous twins can be discordant for AD. Albeit this is still controversial and the literature is sparse, it has been suggested that adverse events in early life, for example, maternal stress and poor maternal and perinatal nutrition, can potentially predispose eventually to AD through epigenetic programing of specific genes/pathways related to AD neurodegeneration. For example, maternal separation for the first 3 weeks of rodent life is shown to result in increase of AD cellular pathways (e.g., APP misprocessing and Tau hyperphosphorylation; see below) followed by synaptic and neuronal damage as well as cognitive deficits in adulthood [40] suggesting the potential impact of early-life stress exposure to the precipitation of AD neurodegeneration later in life. While most current research on epigenetic mechanisms focuses on DNA methylation, one recent study demonstrated that GC, acting via GR, increase the levels of histone deacetylase 2 (HDAC2), an enzyme regulating DNA expression, in the CK-p25 mouse [41]. In general, how early life stressors reprogram the fetal brain and contribute to late-life development of neurodegenerative disorders (e.g., AD) is emerging as an exciting, new research field [42].

Experimental evidence in animal studies indicates that stressful events in early life can impact the etiopathogenesis of another neurodegenerative disorder, Parkinson's disease (PD), which is characterized by both motor and nonmotor symptoms. Depression, anxiety, apathy and interestingly fatigue are common nonmotor features occurring in around 30 to 58% of patients before the onset of motor symptoms in PD patients. In addition, the prevalence of cognitive impairment in PD ranges from 19 to 36% [43]. The cellular mechanisms underlying these nonmotor symptoms in PD may share similarities to AD, particularly with respect to the molecular pathways activated by stress.

Maternal separation was reported to exacerbate motor deficits and nigrostriatal lesion in an experimental model of PD [44]. In an interesting study, pups of female animals, exposed to the bacterial endotoxin lipopolysaccharide (LPS) during pregnancy, showed loss of dopaminergic (DA) neurons. Since loss of dopaminergic neurons as well as related motor deficits is a characteristic feature of PD pathology, the above findings suggest that high LPS levels in mothers might interfere with the development of DA neurons in the fetus, thus enhancing susceptibility to PD [45]. Accordingly, developmental stress may represent the first imprint in the brain and accumulatively with later stressful stimuli to affect nigrostriatal neurochemical reserve and precipitate the PD phenotype [46].

## 3. Chronic Stress and GC as a Risk Factor for AD

AD is a multifactorial neurodegenerative disorder with complex etiopathology. Besides early life stress (see above), accumulating clinical evidence strongly suggests that chronic stress in adulthood as well as elevated GC levels may have a role in the development of AD pathology and related dementia [47, 48]. In fact, high levels of cortisol are commonly found in AD patients' plasma, saliva, and/or CSF [49–53]; AD patients also show higher total daily secretion of cortisol [54]. The potential link between stress/GC and AD described above is strengthened by emerging evidence that stress may advance the age of onset of the familial form of AD [47, 48, 55] and that cortisol levels in AD patients correlate with their memory deficits [56, 57] suggesting a role for GC on AD. Nevertheless, in the absence of longitudinal studies it is not clear from the available evidence as to whether elevated GC secretion is a cause or a consequence of AD disease.

An important brain area in unraveling the interrelationship between stress, elevated GC, and AD pathology is the hippocampus, which is among the first areas affected in AD patients. Hippocampal lesions in AD brain are not only associated with the deficits in declarative, spatial, and contextual memory but could also be responsible for the suggested HPA axis dysregulation and the subsequent overproduction of GC found in AD patients due to the inhibitory role that hippocampus exhibits on HPA axis. Indeed, previous studies from our laboratories (and others) have shown that hippocampal neurons are particularly vulnerable to the adverse effects of stress and GC, their effects being manifested as dendritic atrophy and apoptotic cell death [22, 58]. Moreover, a large number of studies have shown that stress and elevated GC levels affect neurogenesis in adult brain with subsequent impairments of mood and cognitive behavior [59, 60]. More specifically, both acute and chronic exposure stress reduces adult neurogenesis, affecting hippocampal cell proliferation and, in certain studies, survival of newborns [61, 62]. In addition, administration of corticosterone showed the ability of glucocorticoids to damage neurogenesis in adult brain by inhibiting cell proliferation, differentiation and survival [63] while the deleterious effect of stress and/or corticosterone on neurogenesis is GC-dependent [64]. In a vicious cycle, alteration in neurogenesis of adult brain is recently shown to impact on GC negative feedback on the central elements regulating HPA axis activity [65, 66]. Moreover, perturbations in adult neurogenesis may also be related to the cognitive deficits associated with AD whereas contradictory findings support both increases and decreases of neurogenesis in brain of AD patients and Tg animal models [67]. Here, it is also worthwhile noting that stress and GC interfere with hippocampal-prefrontal cortex (PFC) connectivity [68] and dendritic and synaptic plasticity in the PFC, thus disrupting executive functions [58]. These PFC structural deficits are also likely to have consequences for central regulation of the HPA axis providing another neuroanatomical link between HPA axis dysregulation and subsequent GC hypersecretion and AD pathology.

## 4. Impact of Stress and GC on Neurodegenerative Mechanisms of AD

At the molecular level, AD pathology is characterized by amyloid beta (A*β*) that forms deposits (senile plaques) and hyperphosphorylated forms of the cytoskeletal protein Tau that aggregate into neurofibrillary tangles (NFT) [69–71]. A*β* is the proteolytic product of a large transmembrane protein called amyloid precursor protein (APP), which is sequentially cleaved by *β*-secretase (BACE-1) and *γ*-secretase (a complex of enzymes) to generate the production of A*β*; this cellular pathway is often called APP misprocessing. Many studies have demonstrated that the products of APP misprocessing trigger neuropathological processes associated with AD such as synaptic malfunction (including impairment of long-term potentiation), neuronal atrophy and synaptic disintegration and loss [72] as well as mitochondrial dysfunction, oxidative stress, and glial activation [73].

Although still a subject of debate, several studies suggest that A*β* also triggers the abnormal hyperphosphorylation of Tau, NFT formation, and neuronal loss. Moreover, cumulative evidence suggests that the detrimental effects of A*β* are abolished in Tau-KO mice, highlighting the essential mediatory role of Tau protein in the neuro- and synaptotoxic effects of A*β* [73–77]. Further support for an essential role of Tau in the establishment of AD pathology derives from clinical findings that have consistently shown that the cognitive deficits in AD patients correlate better with NFT rather with A*β* deposition* per se*. Moreover, Gómez-Isla et al. [78] demonstrated a strong correlation between neuronal loss in the cerebral cortex and increased NFT burden with disease progression; no such correlation was found with A*β*. In addition, the reduction of hippocampal volume in AD patients correlates better with CSF levels of phosphorylated Tau than with those of A*β* [79].

**Figure 2:**
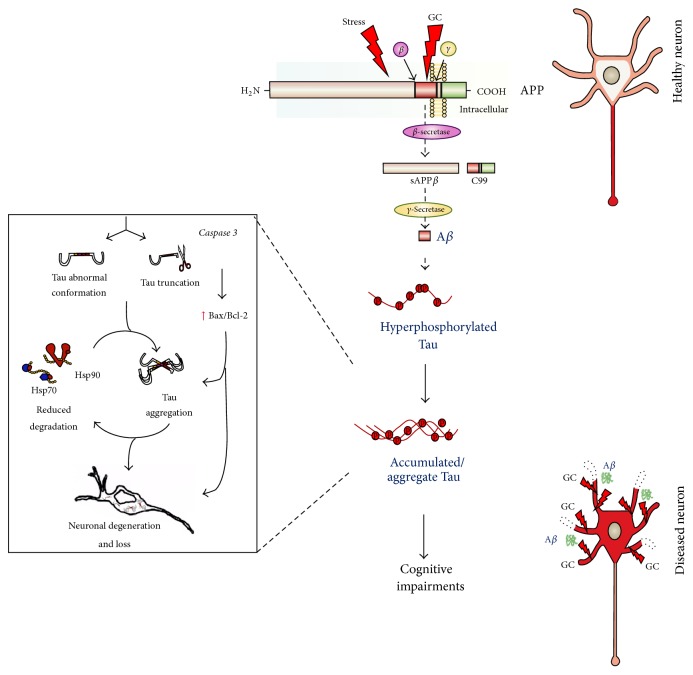
Proposed model through which chronic stress and glucocorticoids (GCs) may contribute to Alzheimer disease (AD) pathology. The model illustrates how chronic stress and high GC levels can trigger AD pathology; the figure is based on experimental evidence obtained in cellular and animal models of AD. Extended exposure to stress/high GC levels activates the amyloidogenic pathway of amyloid precursor protein (APP). This so-called misprocessing of APP involves the sequential cleavage of APP by *β*- and *γ*-secretases, resulting in the generation of toxic amyloid *β* (A*β*). Subsequently, the cytoskeletal protein Tau, which is mainly localized in axons (red in the representation of a healthy neuron), becomes aberrantly hyperphosphorylated, catalyzed by glycogen synthase kinase (GSK3*β*) and/or cyclin-dependent kinase 5 (CDK5). Hyperphosphorylated Tau is trafficked to, and accumulates in, the somatodendritic compartment, where it oligomerizes and forms insoluble aggregates (red in the diseased neuron). In addition, the abnormal conformation adopted by Tau and caspase 3-mediated truncation of Tau is accompanied by dysregulation of the molecular chaperones Hsp90 and Hsp70, which normally serve to promote Tau degradation (left panel). This cascade of events causes neuronal atrophy and loss, followed by cognitive impairments.

The evidence of a causal relationship between stress/GC and AD includes that from studies showing that either high GC levels and/or stress increase the production of A*β* and exacerbate memory deficits in transgenic mouse models of AD [80, 81]. Specifically, chronic immobilization stress in transgenic mice expressing the amyloid precursor protein (APP) V717ICT-100 (a mutation which results in aggressive early onset AD) accelerates the appearance of extracellular A*β* deposits and worsens memory deficits. Similar findings were obtained* in vivo* when young (prodromal) 3XTg-AD mice were treated with the synthetic GC, dexamethasone [80]; the same authors also reported dexamethasone-induced APP misprocessing in the N2A cell line, a finding matched by our own observations in PC12 cells [82]. Further, Green et al. demonstrated that GCs upregulate the transcription of* APP* and *β-secretase*, whose promoters contain a glucocorticoid response element (GRE) [80]. Consistent with the above, our studies in middle aged rats showed that stress and chronic GC drive APP processing towards the generation of A*β* and its precursor molecule (C99), both of which have neurotoxic and cognition-impairing properties [83] (see also Figure 1). The latter changes were accompanied by increases in the levels of *β*-secretase (BACE-1) and Nicastrin, a protein found in the *γ*-secretase complex. Further experiments that attempted to mimic intermittent stressful events that may exert cumulative effects over the lifetime indicated that GC potentiate the APP misprocessing pathway in previously stressed rats receiving A*β*-infusions [83] (see Figure 2).

In addition to triggering the amyloidogenic pathway, high levels of GC and stress can also instigate the aberrant hyperphosphorylation of Tau protein that also characterized AD brain. Among the first reports suggesting a potential connection between GCs and Tau was that from Stein-Behrens et al. [84] who demonstrated that GC exacerbate kainic acid-induced hippocampal neuronal loss with a contemporaneous increase in Tau immunoreactivity. A later study showed that chronic treatment of 3xTg AD mice with dexamethasone leads to the somatodendritic accumulation of Tau in the hippocampus, amygdala and cortex [80].

Supporting those earlier studies, we showed that chronic stress or GC increase the levels of aberrantly hyperphosphorylated Tau in the rat hippocampus and PFC [85] (see Figure 2). Importantly, the hyperphosphorylation occurred at certain Tau epitopes that are strongly implicated in cytoskeletal dysfunction and synaptic loss (e.g., pSer262) [86, 87] and hippocampal atrophy (e.g., pThr231) [88] in AD patients. Here, it is pertinent to note that the extent of phosphorylation at Thr231- and Ser262-Tau correlates strongly with severity of memory impairment, speed of mental processing, and executive functioning in AD patients [89–91]. Although chronic stress and GC treatment exert similar, but not identical, effects on individual Tau phosphoepitopes* in vivo* and* in vitro* [82], the overall evidence points to GC as the key mediator of the AD-like pathology induced by stress. On the other hand, some studies have suggested a role for at least one other stress-related molecule, namely, corticotrophin-releasing hormone (CRH), as deletion of the* CRH receptor 1* gene in mice was found to block the detrimental effects of stress on Tau phosphorylation [92, 93].

As shown at Figure 2, information on the mechanisms underlying stress/GC-induced hyperphosphorylation of Tau is only just beginning to emerge. For example,* in vitro* experiments indicate that the effects of stress/GC are mediated by glycogen synthase kinase 3 (GSK3) and cyclin-dependent kinase 5 (CDK5), both of which have well-established roles in Tau hyperphosphorylation and the subsequent disruption of microtubules, features seen in the AD brain [82]. We now also know that GC exposure increases Tau accumulation by affecting turnover of the protein by reducing its degradation [82]; the latter appears to result from dysregulation of molecular chaperones (e.g., Hsp90 and Hsp70) that are responsible for Tau proteostasis [94] (see Figure 2). Interestingly, both these heat shock proteins also serve to maintain GR in a high affinity state, suggesting that these proteins may be the point at which GC/GR signaling intersects with the cellular machinery that regulates Tau degradation. Using a transgenic mouse that expresses human P301L-Tau (the most common Tau mutation), we recently showed that chronic stress triggers different aspects of Tau pathology in addition to inducing, its aberrant hyperphosphorylation and aggregation of Tau into insoluble forms [94]. Adding to the mechanistic understanding of stress-driven aggregation of Tau, we also showed that chronic stress enhances caspase 3-mediated truncation of Tau at its C-terminal, leading to an abnormal conformation of Tau in the hippocampus (Figure 2). This truncation-dependent misfolding of Tau into an abnormal conformation is known to facilitate nucleation and recruitment of other Tau molecules into neurotoxic aggregates [95, 96] before NFT are formed [95, 97, 98].

It is interesting to note that chronically elevated GC secretion, usually in response to stress, is a major cause of major depressive illness [99]. In light of the increasing volume of data implicating high GC levels in AD, it is important to consider that epidemiological studies implicate depression as a risk factor for the development of AD; this is supported by the observation that previously depressed subjects have increased amyloid plaque and NFT loads [100]. Different studies have in fact sought to discriminate between subjects undergoing normal aging from those suffering from depression or AD through the measurement of the various APP cleavage products [101–104]. While much remains to be discovered about the potentially important role of depression in AD pathology, it is interesting to note that antidepressant drugs, whose actions often involve reductions in GC secretion, inhibit the proteolytic cleavage of APP into amyloidogenic products [104, 105].

Lastly, it deserves mentioning that a recent epidemiological study found that the prevalence and incidence of dementia in war veterans suffering from posttraumatic depression (PTSD) is twice as high as that in age-matched PTSD-free subjects [106]. While PTSD is a condition quite distinct from major depression, these findings hint at the important influence lifetime stressful experiences can have on mental health, possibly through epigenetic mechanisms. The findings are also interesting since PTSD patients usually show hypoactivity of the HPA axis (versus hyperactivity in depression), suggesting that just a single—but major stressful—event involving transient GC hypersecretion can have long-lasting neuropathological consequences.

### 4.1. Inflammation and AD: Role of GCs?

Chronic inflammation is one of the central pathological features of AD with reactive microglia and astrocytes surrounding senile *β*-amyloid plaques observed in both postmortem AD brain and animal models [107, 108]. Evidence from human studies suggests that glial activation is an early event; thus inflammatory markers are present in mild cognitive impairment cases that eventually progress to AD [109]. Thus proinflammatory cytokines produced by activated glia in response to amyloid fibrils would be expected to activate HPA axis and increase GC levels.* In vitro* studies clearly show that A*β* can be taken up through phagocytosis in microglia and thereafter degraded [110, 111]; thus, in AD setting, microglial likely have a beneficial role early in pathology. However, elevation of proinflammatory cytokines such as IL-1*β* may also participate in mood disorders such as depression [112] in AD.

The importance of immune-related responses in the emergence of A*β* burden, tau pathology, and dementia is gaining momentum as molecular comprehension of their actions is increasingly unraveled by human genetic and animal studies. Recent genome-wide association studies have identified variants in at least 16 genes involved in microglia/macrophage functions as risks for developing AD [113]. Among them, *ε4* allele of* APOE* gene is a known strong risk factor, accelerating the age of onset of AD. APOE is produced by both microglia and astrocytes; it regulates not only lipid and A*β* metabolism but also microglial chemotaxis and proinflammatory cytokine expression [114]. Recently, another strong link was found between variants in* TREM2* gene and AD. TREM2 is specifically expressed in myeloid cells where it promotes phagocytosis whilst inhibiting cytokine production [115]. These and most other GWAS genes identified [113] are involved in aberrant microglial/macrophage responses with regard to A*β* clearance and spread of Tau pathology.

In addition to genetic susceptibility, prolonged exposure of A*β* affects microglial functions. Thus, crucial microglial functions such as motility and phagocytosis were impaired in APP/PS1 mice [116]; also in these mice the levels of A*β* receptors (SRA, CD36, RAGE) and A*β* degrading enzymes (neprilysin, MMP9) were decreased with concomitant increase in proinflammatory cytokines TNF-*α* and IL-1*β* [117]. Age, a primary risk factor for AD, is also an important contributor to dysfunction of innate immune responses. Microglial dystrophy and fragmentation observed in aging brain [118] occur before the appearance of abnormal Tau suggesting dysfunctional microglia could contribute to appearance of Tau pathology.

Chronic stress through GCs is known to prime and augment neuroinflammatory processes in the cortex and hippocampus upon subsequent proinflammatory challenges such as LPS [119, 120]. Peripheral infections and stress are both known to affect the activation state of microglia and in AD pathology both could have detrimental effects on the functions of microglia. There is little known on how glucocorticoids influence glial functions during prodromal to emergence and progression of AD pathology. It would be important to understand whether GC through GR has any role in A*β* degradation in astrocytes or myeloid cells.

## 5. Role of Glucocorticoids in Onset and Progression of Parkinson's Disease

Parkinson's disease (PD) is a complex systemic and progressive neurodegenerative disease associated with both motor and nonmotor symptoms. The cardinal motor symptoms such as akinesia, resting tremor and rigidity mostly arise from preferential and substantial loss of dopaminergic neurons (50–60%) in the substantia nigra pars compacta (SNpc) with significant dopamine depletion in the sensorimotor striatum. The nonmotor symptoms include olfactory dysfunction and sleep behavior disorder as well as mood changes and cognitive impairment as discussed above. One principle histopathological feature is the presence of Lewy bodies (LBs), which are proteinaceous inclusions containing mainly structurally altered presynaptic protein, alpha-synuclein, which, as recent evidence shows, plays a central role in PD pathology. Alpha-synuclein LB deposition was used by Braak et al. [121] as a principle pathological marker to monitor the progression and severity of PD. PD is believed to originate from olfactory nucleus and autonomic nervous system progressing in an ascending manner to many brain regions such as substantia nigra, striatum, raphe, locus coeruleus, hypothalamic nuclei, hippocampus, amygdala, and cerebral cortex accounting for both motor and nonmotor symptoms [121–123]. Thus, for example, PD patients with cortical LBs also suffer from dementia and visual hallucinations [124].

While several gene mutations have been identified in familial forms of PD, the majority of PD cases are sporadic and of unknown etiology. Nevertheless, significant advances in the last decade on PD genetics, particularly genome-wide association, as well as pathophysiological mechanisms in various PD model systems, have contributed much to our comprehension of PD. Cellular processes such as oxidative and nitrative stress, mitochondrial dysfunction, and deregulated intracellular calcium levels as well as damaged proteostasis related to alpha-synuclein aggregation are the most studied and relate to dopamine neurodegeneration [125].

As in AD patients, the HPA axis is likely dysregulated in PD patients. Specifically, previous studies [54, 126–128] including our own work [129] show that plasma cortisol levels are significantly higher in idiopathic PD patients compared to control subjects; however, these high levels do not correlate to disease duration or to L-3,4-dihydroxyphenylalanine (L-DOPA) treatment. Interestingly, the diurnal pattern of cortisol secretion in PD patients, in particular the normally quiescent nocturnal cortisol secretory pattern, is affected [54].

## 6. The Neurodegenerative Potential of Altered GC Levels in PD Pathology

**Figure 3:**
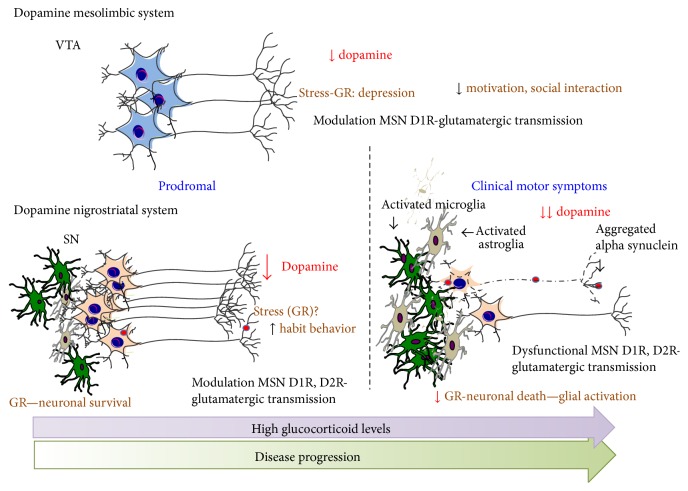
Putative impact of elevated GC levels on GR function in nigrostriatal and mesolimbic dopaminergic systems in PD. Stress-level elevation of GCs may be an early feature of PD, potentially impacting both motor and nonmotor dopaminergic systems. Mesolimbic dopaminergic circuitry is likely affected through structural and functional changes occurring in D1R MSNs. These changes lead to depression and reduced motivation and social interaction which are key prodromal features of PD. Dopaminergic neurons in VTA are relatively spared in PD. In the nigrostriatal system, high levels of GCs initially protect dopaminergic neurons of substantia nigra through dampening the immune responses, namely, mediated by activated microglia and astrocytes. In the putamen, high stress levels of GCs through GR augment habit learning and may act to prevent the appearance of motor symptoms. With disease progression, GR function is affected, leading to chronic glial and immune activation, which exacerbates dopamine neurodegeneration with significant dopamine depletion in the striatum. Changes in GR activity may also affect striatal D1 and D2R MSNs further participating in the appearance of clinical motor symptoms.

Chronically elevated GC levels in PD patients suggest that HPA regulated-stress responses may impact PD pathology. Indeed, the role of stress was proposed as one of the underlying causes of PD as clinical reports show that stress triggers the appearance of PD symptoms or exacerbates the motor symptoms [130–132]. The role of stress in PD is supported by few experimental studies such as food deprivation and tail-shock and maternal separation aggravate motor deficits in the 6-hydroxydopamine (6-OHDA) PD model (6-hydroxydopamine local injections lesions the nigrostriatal pathway) [133]. In combined chronic stress exposure with 6-OHDA lesion, stress was shown to worsen the 6-OHDA-driven motor deficits, aggravate the neurodegeneration of nigrostriatal system, and completely block compensatory recovery of motor tasks [131, 134]. The precise actions of high GC levels in motor control following nigrostriatal lesions are yet not known. Analysis of GR expression in PD brains revealed that GR levels were reduced in the SNpc and augmented in the putamen, compared to age-matched control subjects; similar results were found in MPTP- (1-methyl 4-phenyl 1,2,3,6-tetrahydropyridine-) treated mice [129]. GCs are known to profoundly modulate dopaminergic neurotransmission. The role of GC on the limbic arm of the dopaminergic circuitry related to reward and motivation as well as neuropsychiatric diseases has been extensively investigated (see below). Thus, from its known roles in mesolimbic circuitry, it has been postulated that GR also likely affect motor automated or habitual skills of the sensorimotor circuitry in the striatum by influencing NMDA/AMPA receptor functions in D1 and D2 receptor-medium spiny neurons (Figure 3). Indeed, it has been shown that chronic stress leads to opposing structural changes in the limbic/associative and sensorimotor striatal circuitry with atrophy in the former and hypertrophy of sensorimotor striatum, leading to habit behavior [135]. In addition, the roles of both glucocorticoids and noradrenaline were recently reported in habit memory [136]. It is possible that GR-mediated changes in the putamen during the prodromal stage of PD play a role in preventing the appearance of motor symptoms, culminating in dopamine depletion and death of dopaminergic neurons in the substantia nigra.

Altered stress responses most likely play an important role in nonmotor PD symptoms, particularly anxiety, depression, and mild cognitive impairment, which often precede motor symptoms. Interestingly, there is also evidence in PD for lower novelty-seeking and high harm avoidance personality traits with anxiety-associated symptoms [43, 137]. These observations suggest that, in the initial disease stage, stress-related alterations in GC-GR activity could impact both the motivation/cognitive-associated dopaminergic as well as nondopaminergic (serotonergic and noradrenergic) neuronal circuitry. This would also implicate dopaminergic neurons in the ventral tegmentum area (VTA), which although relatively spared in PD are well-known to regulate reward and aversion by stress and have been implicated not only in addiction but also depression involving the transcriptional factor CREB and BDNF [138–141]. On the other hand, dorsolateral dopamine neurons in the SN (vulnerable in PD) were shown to respond to tasks involving working memory [142]; thus, their demise could explain, in part, the cognitive deficits observed in PD. Studies on the dopaminergic transmission during stress have revealed the complexity of the system. In fact, firing patterns of dopamine neurons in VTA correlated with depressive-like behaviors in mice, although the effect appears to depend on the stress paradigm used to induce the depressive-like behavior [139, 143]. Electrophysiological evidence implicates changes in both D1R and D2R-medium spiny neurons (MSNs) in the ventral striatum [144], but the depressive-behaviors seems to preferentially affect D1R MSNs [145] (Figure 3). Glutamatergic receptors, NMDA and AMPA receptor functions were shown to be also altered in the D1R MSNs, notably NMDAR-dependent LTD, reduced AMPA/NMDA receptor ratio and increased endocytosis of AMPA receptors [146].

## 7. Role of Glucocorticoid Receptors in Inflammation-Induced Neurodegenerative Processes and Nonmotor Symptoms in Parkinson's Disease

Accumulating evidence points to inflammation resulting from chronic activation of innate and adaptive immune cells as playing an important role in both neurodegenerative processes and in nonmotor symptoms of PD. Using radiolabeled ligand ^11^C-PK-11195 for translocator protein, Positron Emission Tomography (PET) studies in PD patients revealed an early activation of microglia in many brain regions including basal ganglia and midbrain [147, 148]. Furthermore, postmortem studies as well as analyses of serum and cerebrospinal fluid from PD showed high levels of proinflammatory mediators such as TNF-*α*, IL-1*β*, iNOS, IFN-*γ*, and COX-2 [149]. In line with observations in PD patients, presence of inflammatory mediators and glial reactivity in striatum and substantia nigra is a key feature in many of the experimental animal models of PD [150]. Evidence from recent genome-wide studies points to involvement of the immune system in the etiology of idiopathic PD. A number of susceptibility loci identified relate to genes expressed in immune cells such as HLA-DQB1, LRRK2 or BST-1 [151, 152]. In addition, identified PD risk factors [such as age, environmental toxins (e.g., heavy metals or pesticides,) traumatic brain injury, and bacterial or viral infections] activate immune responses in periphery and brain.

### 7.1. GR Regulation of Inflammation Important for Dopamine Neuronal Survival

Activated microglia functioning as innate-immune competent cells are likely involved in releasing the above inflammatory molecules, thereby inducing dopamine neurodegeneration. Indeed, the important role of these proinflammatory mediators in promoting degeneration of dopaminergic neurons of substantia nigra was demonstrated using mice with specific knockout of these genes [153–156]. Many of the proinflammatory mediators found in PD patients are transcriptional targets of GR. The synthetic analogue of GCs, dexamethasone, was shown to attenuate dopamine neuronal loss by precluding activated microglia from releasing toxic inflammatory molecules [157, 158]. In adrenalectomized mice (lacking endogenous production of GCs), dopamine neuronal loss was augmented following MPTP intoxication indicating that endogenous GCs do play a role in protecting dopamine neurons [159]. Examination of GR in microglia revealed an increase in nuclear localization of GR following MPTP treatment in mice, which coincided with a rise in systemic corticosterone levels, indicating that GR is activated in microglia during the degeneration of dopamine neurons [129]. The unequivocal evidence that GR in microglia normally protects dopamine neurons was provided by experiments with mice in which the GR gene was selectively deleted in microglia/macrophages. MPTP treatment in these mice resulted in increased dopamine neuronal loss as well as increased microglial activation and expression of proinflammatory mediators [129]. Indeed, the absence of GR in microglia resulted in sustained activation of NF-*κ*B as was shown in these microglial GR mutants. The above findings have a significant relevance for PD pathogenesis as nuclear expression of p65 subunit of NF-*κ*B, indicative of transcriptional activity, was found in the substantia nigra microglia of PD postmortem [160].

Inflammatory reaction mediated by immune-competent cells such as microglia is normally a very tightly regulated process of limited duration. It is very likely that the processes involved in the regulation of glial immune responses including the expression and secretion of inflammatory mediators are compromised in PD and also AD resulting in a chronic inflammatory state with sustained activation of glia spanning many years. One likely factor contributing to dysfunction of glial immune responses is aging. Immune-regulatory processes are compromised in aging (immunosenescence) and also during chronic stress [161] where there is an increased susceptibility to infections as well as proinflammatory cytokine production [162]. In aging, microglia show enhanced sensitivity to inflammatory stimuli, a process called “priming” which could be also induced by chronic stress and a dysregulated HPA axis. In this regard, there are several studies showing that chronically elevated GCs levels in response to different stressors cause proinflammatory cytokine production and sensitization or “priming” of microglia. Importantly, subsequent inflammatory or toxic stimuli result in aggravation of neuronal injury [119, 120, 163]. Moreover high and sustained GCs can exacerbate inflammation because of GC resistance whereby GR activity is affected. Thus it is plausible that GR transcriptional activity regulating inflammatory response of microglia is compromised in AD and PD patients who display persistently high GC levels.

### 7.2. GR, Inflammation and Nonmotor PD Symptoms

Recent experimental evidence shows that glia and peripheral immune cells are activated upon chronic psychogenic stress and that their actions are important in mood and behavior [164–167]. Glial production of potent proinflammatory cytokines such as TNF-*α*, IL-6, and INF-*γ* are implicated in depression through stimulation of the kynurenine pathway (shift of serotonin synthesis from tryptophan to kyneurin) in activated astroglia, microglia, and infiltrating peripheral immune cells. Kynurenine, produced from tryptophan by activation of indoleamine 2,3-dioxygenase (IDO), can be further converted to kynurenic acid or quinolinic acid, the latter affecting the function of both monoaminergic and glutamatergic neurons. Quinolinic acid toxicity with increased glutamate release results in lipid peroxidation and nitrative stress [168, 169] Evidence shows that the kynurenic acid/tryptophan ratio is altered in CSF and serum in PD patients [170].

Another means by which glial activation and proinflammatory cytokines promote mood anomalies in PD is through reducing neurogenesis in hippocampal subgranular zone, thus affecting hippocampus-mediated regulation of mood and cognition [171].

## 8. Conclusion

Clinical and preclinical studies suggest that chronic stress/elevated GC levels may be an etiological factor in the development and progression of both AD and PD pathologies. Growing evidence indicates that the pathological manifestations of chronic stress include neuronal and synaptic atrophy/malfunction as well as immunosuppression, but our understanding of the underpinning mechanisms is still poor and calls for more research not only to identify therapeutic inroads but, also, preventative measures or ways to delay onset of disease.
